# Microstructures and Properties of Laser Cladding Al-TiC-CeO_2_ Composite Coatings

**DOI:** 10.3390/ma11020198

**Published:** 2018-01-26

**Authors:** Xing He, Dejun Kong, Renguo Song

**Affiliations:** 1 School of Materials Science and Engineering, Changzhou University, Changzhou 213164, Jiangsu, China; hx_ccu@163.com; 2 Jiangsu Key Laboratory of Materials Surface Science and Technology, Changzhou University, Changzhou 213164, Jiangsu, China; djkong@cczu.edu.cn; 3 Jiangsu Collaborative Innovation Center of Photovolatic Science and Engineering, Changzhou University, Changzhou 213164, Jiangsu, China; 4 School of Mechanical Engineering, Changzhou University, Changzhou 213164, Jiangsu, China

**Keywords:** laser cladding, Al-TiC-CeO_2_ composite coating, microstructure, property

## Abstract

Al-TiC-CeO_2_ composite coatings have been prepared by using a laser cladding technique, and the microstructure and properties of the resulting composite coatings have been investigated using scanning electron microscopy (SEM), a 3D microscope system, X-ray diffraction (XRD), micro-hardness testing, X-ray stress measurements, friction and wear testing, and an electrochemical workstation. The results showed that an Al-Fe phase appears in the coatings under different applied laser powers and shows good metallurgical bonding with the matrix. The dilution rate of the coating first decreases and then increases with increasing laser power. The coating was transformed from massive and short rod-like structures into a fine granular structure, and the effect of fine grain strengthening is significant. The microhardness of the coatings first decreases and then increases with increasing laser power, and the maximum microhardness can reach 964.3 HV_0.2_. In addition, the residual stress of the coating surface was tensile stress, and crack size increases with increasing stress. When the laser power was 1.6 kW, the coating showed high corrosion resistance.

## 1. Introduction

S355 is a special steel commonly used for offshore platforms. Because of the harsh environment, the underwater structure must be resistant to corrosion by sea water and marine life, endure long periods of service, and exhibit high strength and resistance to wear, corrosion, cracking, and other issues. Thus, higher strength, corrosion resistance, wear resistance and other characteristics are necessary [1]. At present, long-term corrosion protection of offshore platforms generally takes the form of improved surface coating technology, but maintenance is very difficult and expensive. Therefore, the ideal coating would provide long-term preservation of the underlying material, and must be a high-performance composite coating material. Minimizing the number of coating repairs and thus extending the maintenance cycle is very desirable. Laser cladding is an advanced surface modification technique that adds cladding powder to the substrate surface by different packing methods. After laser irradiation, the coating is fused with the thin layer on the surface of the substrate and rapidly solidified, becoming metallurgically bonded to the substrate. This surface hardening method can significantly improve the wear, corrosion, fatigue, and oxidation resistance of the substrate [2,3,4,5,6,7]. Compared with more traditional methods, laser cladding is safer, has a smaller affected heat zone, does not produce pollutants, and the resulting surfaces will not harm the marine environment. At the same time, the laser cladding temperature is very high, and using a laser heat treatment offers the advantages of limited thermal deformation and precise control of the coating thickness [8,9,10,11,12,13]. Because Al coatings have a low electrode potential and good oxidation resistance, Al coatings on various metal substrates show good anti-corrosion performance and improved physical and chemical properties. High-power laser cladding can induce an Al-Fe compound reaction at the coating interface, resulting in the formation of metallurgical bonding to improve the coating resistance to corrosion from sea water [14,15]. Laser cladding Al coatings have become a mature protective technology for offshore steel facilities. A properly enclosed Al coating exhibits excellent corrosion protection of the underlying steel structure in the splash zone at both normal and elevated temperatures [16,17]. Based on the current level of technology, a 200-μm-thick Al cladding coating is expected to last for more than 30 years in the splash area for steel structures. Therefore, the preparation of laser cladding Al coatings has become a hot topic for research in the field of long-term heavy corrosion protection [18,19]. At present, Yang et al. [20] have prepared an Al-TiC in situ composite coating fabricated by low power pulsed laser cladding on AZ91D magnesium alloy. The results show that the wear resistance and corrosion resistance of the coating are enhanced. Chao et al. [21] have prepared Al_x_CoCrFeNi high entropy alloys on a high-temperature stainless steel substrate by laser deposition cladding. The results show that the HEA claddings displayed an evolution of crystal structure from FCC to FCC + BCC and BCC accompanied by an increase in microhardness. The increased Al content also resulted in reduced microstructural stability of the coatings. Wang et al. [22] have shown that CeO_2_ can refine grains, reduce crack initiation and improve cladding quality. Although there has been much research on laser cladding Al-based composite coatings at home and abroad, currently, the application of laser cladding technology to the preparation of high-performance Al-TiC-CeO_2_ composite coatings on offshore steel surfaces is rare. Therefore, this paper reports on the use of laser cladding technology to prepare high-performance Al-TiC-CeO_2_ composite coatings on S355 offshore steel and studies of the resulting surface morphologies, chemical element distributions, and phase compositions of Al-TiC-CeO_2_ composite coatings using SEM, EDS, and XRD. The hardness, wear resistance, residual stress, and corrosion resistance of the coatings were tested and analyzed using a digital microhardness tester, friction and wear tester, X-ray stress tester, and an electrochemical workstation. This paper will focus on analyzing the effects of laser power on the Al-TiC-CeO_2_ composite coating microstructure and properties, which provides an experimental basis for the application of Al-TiC-CeO_2_ composite coatings on offshore platforms.

## 2. Experimental

**Figure 1:**
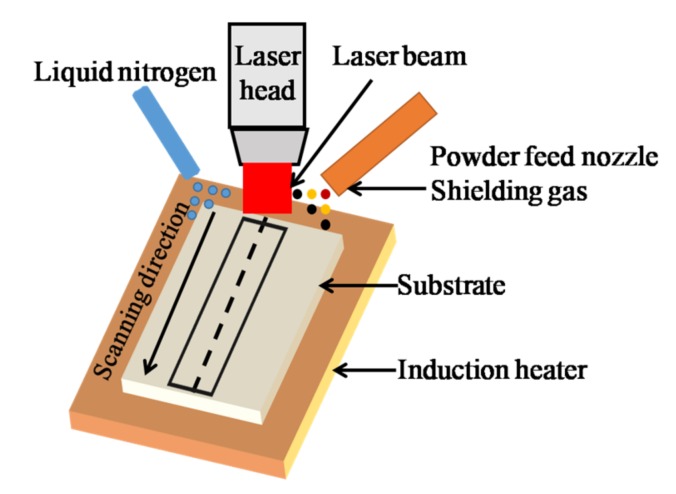
Schematic of laser cladding process.

The spraying materials used were Al, TiC, and CeO_2_ with different ratios of powdered Al and TiC with a mass ratio of 5:1 after adding 0.6% CeO_2_. In order to guarantee the flowability of powder, the particle sizes of Al, TiC, and CeO_2_ were in the range of 20–100 μm, which is considered to be too big enough to agglomerate [23]. The powders were mixed and milled using a QM3SP04L type planetary ball-milling machine to ensure the uniformity of the resulting powders. The experimental material and contrast material was European standard S355 structural steel, and the mass fractions are listed in Table 1. The substrate was cut into rectangular cubes of size 60 mm × 30 mm × 5 mm with a wire cutter. Before the experiment, samples were ground with sandpaper and then washed repeatedly with alcohol and acetone. The laser cladding equipment including an all solid-state laser, and argon gas was used as the power source for powder feeding to realize synchronous powder spraying. The liquid nitrogen cooling system on the worktable provided simultaneous cooling. A schematic of the laser cladding process is shown on Figure 1, and the technological parameters are listed in Table 2. After the cladding experiment, coating I with a laser power of 1 kW, coating II with a laser power of 1.2 kW, coating III with a laser power of 1.4 kW, and coating IV with a laser power of 1.6 kW were obtained. The cladded specimen was then cut into rectangular blocks 10 mm × 10 mm × 3 mm in size, and the surface of the coating was abraded with sandpaper and then polished with Al_2_O_3_. The surface and interface morphologies of the obtained coatings were observed using a field emission scanning electron microscope (FE-SEM). The coatings were sputtered with a thin layer of Au prior to SEM inspection. The morphologies of the surface and interface magnified 3000 times and 50 times, respectively. The elements on surface were analyzed using the SEM associated energy dispersive X-ray spectrometer (EDS), and the morphologies and crack formation on the composites were analyzed using a VHX-6000 type 3-D microscope system. The phases and residual stresses of the coatings were analyzed using XRD and an X-ray stress tester. The technological parameters used for measurement of the residual stress were as follows: fixed peak method for cross correlation, using a Co target, Co-Kα1 radiation source, Prague crystal face of (400), incident angles of 0°, 25°, 35°, and 45°, stress constant of −130 MPa/°, 2θ scan start and termination angles of 155–145°, 2θ scanning step of 0.10°, counting time of 0.50 s, X-ray light tube high voltage of 22 kV, and an X-ray light tube current of 6.0 mA. The microhardness of the coating was measured with an HMV-1T digital microhardness tester with the following conditions: a loading of 200 g and a loading time of 15 s. Measurements were taken every 100 μm from the surface of the cladding to the substrate; at each point in the cross-section, the hardness was measured 3 times at the same level of depth and the results were averaged. Friction and wear testing were conducted using a CFT-1 surface tester. The grinding material used was 45# steel at a loading of 200 g and a motor speed of 500 r/min, using a reciprocating sliding mode, the wear scar radius was 3 mm and the running time was 30 min. The wear medium was air and the measurement was completed using a BT25S electronic analytical balance to measure the weight loss. The electrochemical tests were performed on a CS350 type electrochemical workstation, which was immersed in a 5% NaCl solution for 30 min before the immersion corrosion test where the pH range of the NaCl solution was 6.5 to 7.2, the scanning rate was 1 mV/s, the sampling frequency 0.5 Hz, the measuring temperature 25 °C, and the reference electrode type was mercury/calomel-saturated KCl with a test time of 1800 s.

## 3. Results and Discussion

### 3.1. XRD Analysis of Coating Surfaces

**Figure 2:**
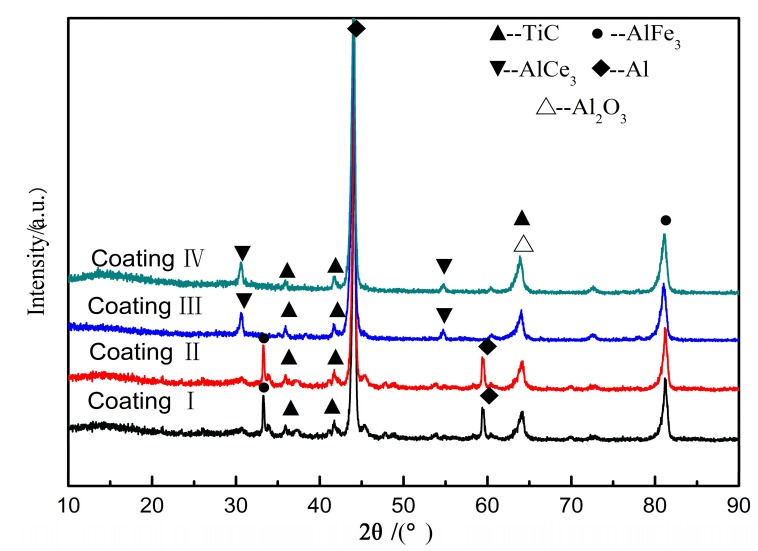
XRD patterns of cladding layer under different laser powers.

The XRD patterns obtained from the coating surfaces are shown on Figure 2. The highly crystalline diffraction peaks of the α-Al phase at 44° and AlFe_3_ peaks at 82° were detected in coatings I, II, III and IV. Also, AlFe_3_ peaks at 34° were detected in coatings I and II. The existence of AlFe_3_ phase suggested that the substrate reacted with powders during laser cladding at high temperature. This result showed that the elements of Al and Fe not only diffused but also formed a new phase in the coating so that a metallurgical bonding between the coating and the substrate occurred, thus enhancing the bonding strength of the interface. At the same time, peaks corresponding to the reinforcement phase TiC were detected at 38°, 42° and 65°, respectively. The smaller crystalline diffraction peak associated with Al_2_O_3_ appears at 65°, indicating that there may be slight oxidation of the coating during the cladding process. Additionally, AlCe_3_ peaks appeared in coating III and IV at about 30° and 55°, but did not appear in coating I or II. This may be due to the fact that less energy is accumulated on the surface of the coating and there is little convection in the melting pool at lower power, thus insufficient melting occurs and the solidification time is too short. As a result, CeO_2_ cannot react with Al in the melting pool, so AlCe_3_ phase has not been detected in coatings I and II.

### 3.2. Morphologies and EDS Analysis of Coating Interfaces and Surfaces 

#### 3.2.1. Morphologies and EDS Analysis of Coating Interfaces

**Figure 3:**
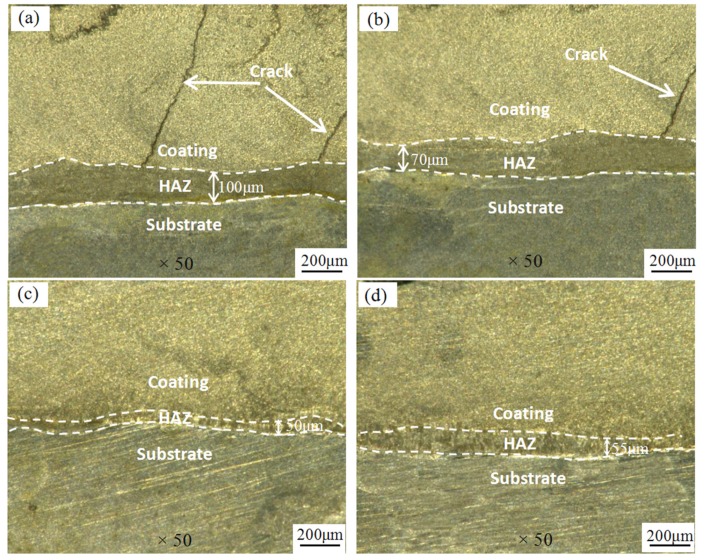
The cross-sectional optical micrographs of coatings, (**a**) Coating I; (**b**) Coating II; (**c**) Coating III; (**d**) Coating IV.

**Figure 4:**
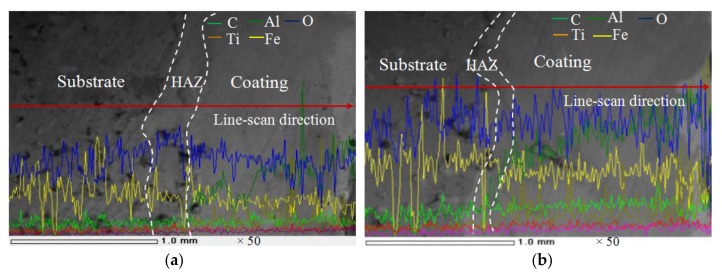

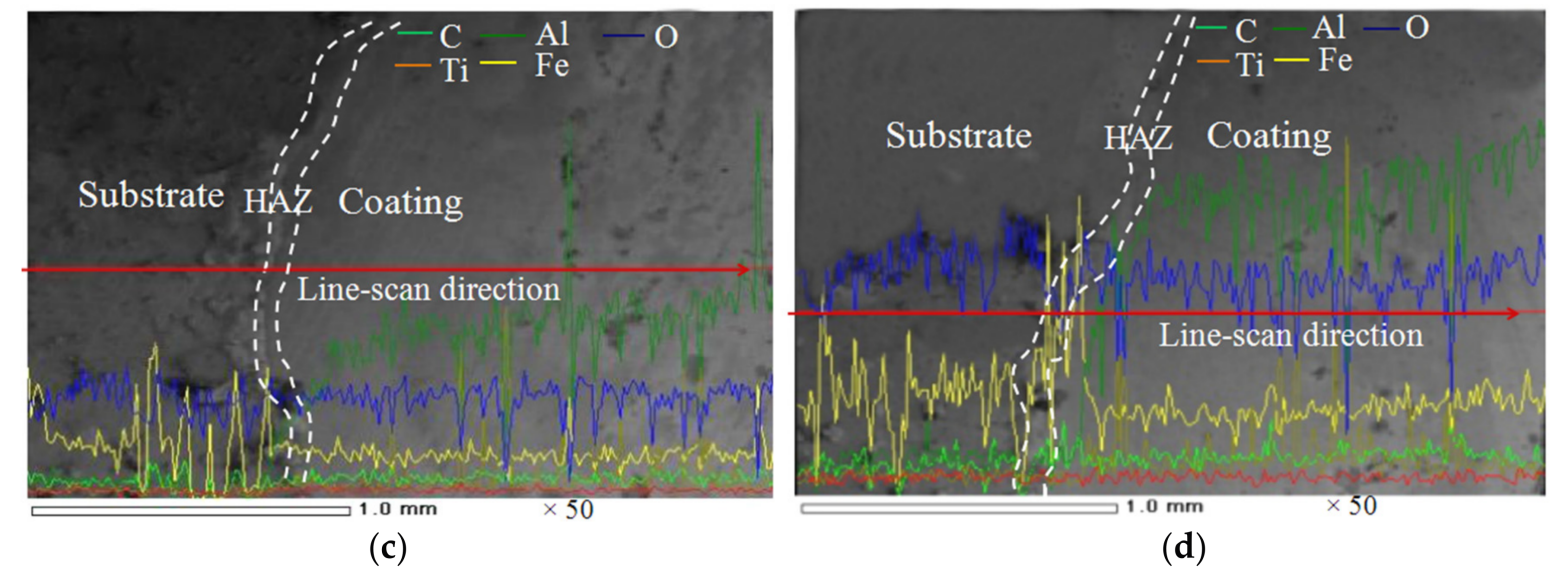
SEM image and EDS line-scan of coating cross-section, (**a**) Coating I; (**b**) Coating II; (**c**) Coating III; (**d**) Coating IV.

The cross-sectional optical micrographs of coatings are shown on Figure 3. It can be seen that the coating are divided into three regions in sequence: cladding layer, heat affected zone (HAZ), and substrate. The HAZ thickness of coatings I, II, III and IV were about 100 μm, 70 μm, 50 μm and 55 μm, respectively. There were obvious cracks on the coatings I and II. The SEM morphologies of the surface coatings and some associated line scans are shown on Figure 4. The main elements at the interfaces are C, O, Al, Ti, and Fe, where O is an impurity element. The Al content increases gradually from the HAZ, while the C and Ti elemental distribution is steady. Fe was detected in the coating, indicating that elements in the matrix diffused into the coating to form a metallurgical bond with the coating. The coating II contains more O, indicating that the coating is more heavily oxidized, while the elemental C and Ti distributions are steady, but the coating still has a small amount of distributed Fe, indicating that the coating and the substrate have formed a metallurgical bond, as shown on Figure 4b. The O content of coating III decreases while the Al content increases gradually. The C and Ti contents are smoothly distributed and a trace amount of Fe has diffused into the coating and formed a better metallurgical bond, as shown on Figure 4c. The O and Fe content of coating IV is less distributed, the Al content is further increased, the distribution of C and Ti is uniform and stable, and the metallurgical bond between the coating and the substrate has been formed, as shown on Figure 4d.

Because the dilution rate directly affects the performance of the coating [24], the dilution rate λ is expressed by the Formula (1):

ë = h/(H + h) × 100%(1)

where H is the coating thickness and h is the melting depth of substrate. The dilution rates of coatings I, II, III and IV can be calculated as 9.09%, 6.54%, 4.76% and 5.21%, respectively. It is generally considered that the dilution rate of the coating is better at 5% [25].

#### 3.2.2. Morphologies and EDS Analysis of Coating Surfaces

**Figure 5:**
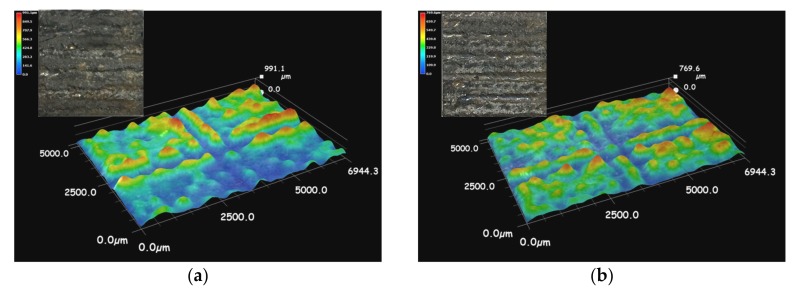

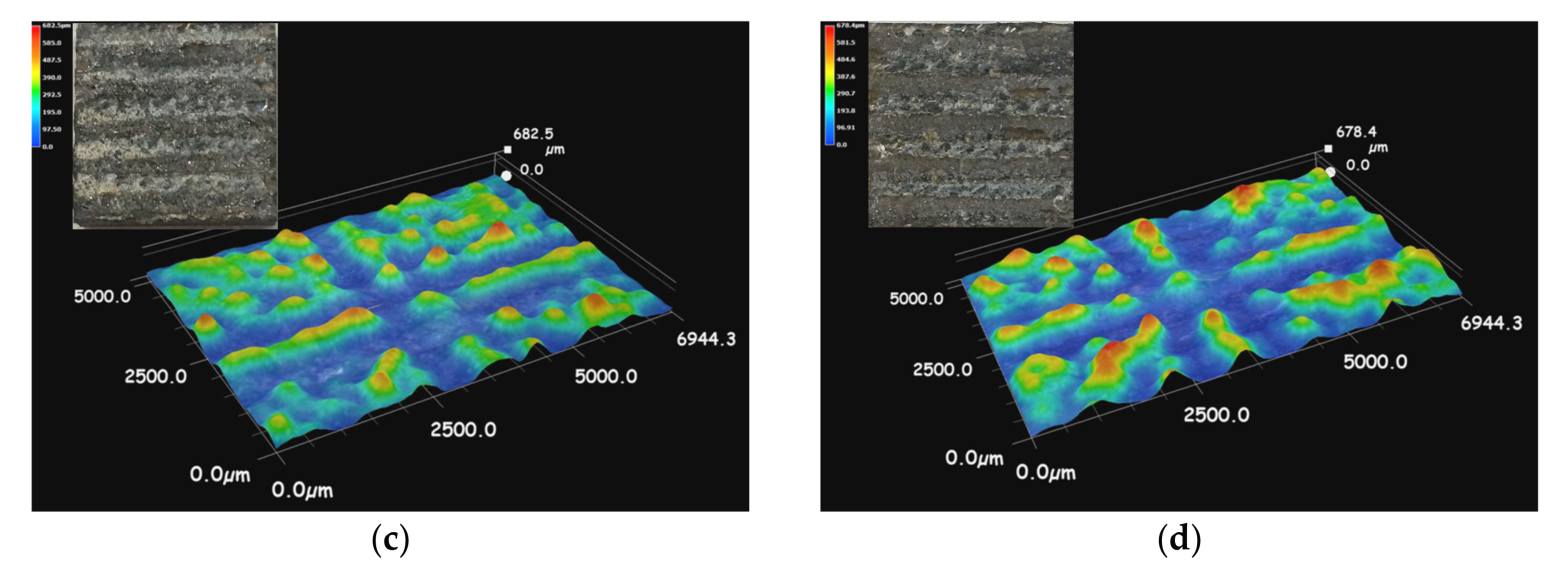
The surface geometry of the coatings: (**a**) Coating I; (**b**) Coating II; (**c**) Coating III; (**d**) Coating IV.

The surface geometries and its corresponding macro photos of the coatings are shown on Figure 5. Rippling waves are formed on the coated surfaces of four different powder samples. The rippling waves observed in coating I reach a height of 991.1 μm, and the surface roughness is large, as shown on Figure 5a. The rippling waves in coating II are 769.6 μm high, as shown on Figure 5b, while the rippling waves in coatings III and IV are 682.5 μm and 678.4 μm high, respectively. The surfaces of the coatings are relatively smooth, as shown on Figure 5c,d. The formation of the ripples could be attributed to the decrease in the fluidity of the molten pool. These formed ripples not only reduce the effective thickness of the coating but also result in stress concentrations in the rippling areas and consequently lead to the formation of cracks [26]. In summary, when the laser power was lower, crack susceptibility and roughness of the coatings became more severe.

**Figure 6:**
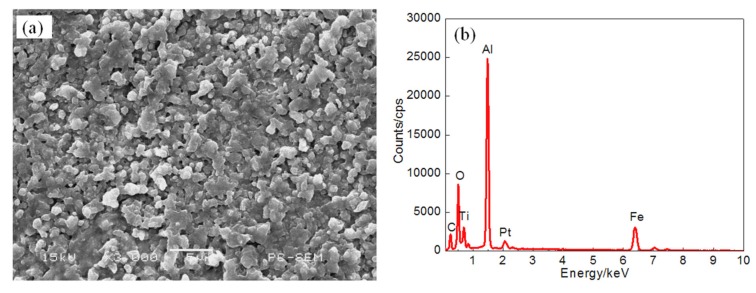

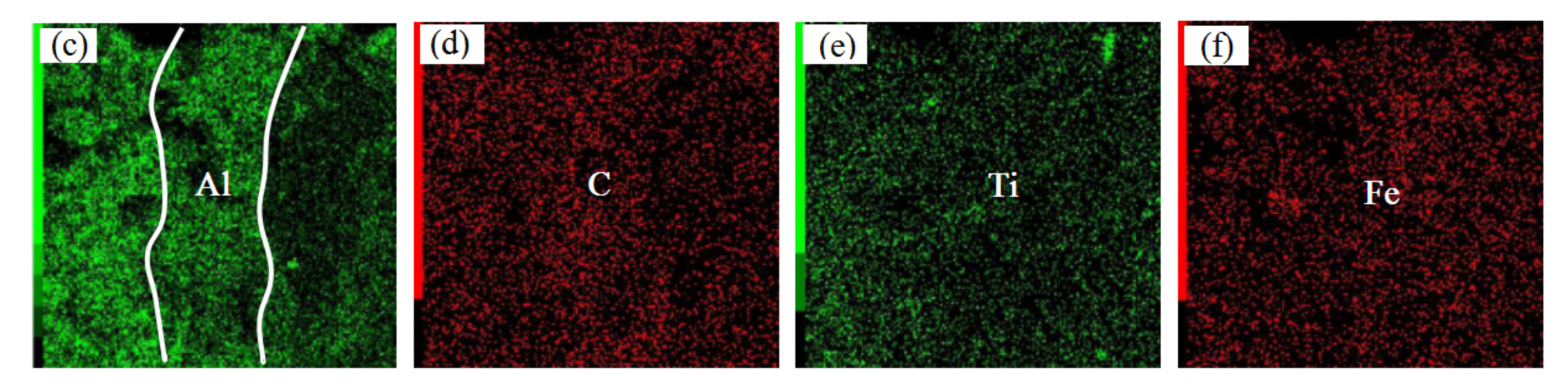
The surface SEM images (**a**) and EDS analysis result (**b**) and element content of Plane scan ((**c**) Al content (**d**) C content (**e**) Ti content (**f**) Fe content) of Coating I.

Figure 6 shows the surface morphology of coating I prepared with a laser power of 1 kW and the EDS plane scan analysis results. At lower power, the surface of coating I mainly consists of a continuous α-Al phase, AlFe_3_ and Al_2_O_3_. The inner layer of the coating is mainly composed of short, massive, rod-like structures. The reinforcing phase TiC is scattered on the surface and the surface is not smooth. The coating surface also contains a large number of irregularly shaped pores, as shown on Figure 6a. Because of the large differences between the thermophysical coefficients of TiC and the substrate, the thermal stress easily occurs under the action of the laser beam. The results of the coating EDS plane scan are shown on Figure 6b. There is a small amount of Pt detected on the coating surface, which may be due to the sputtering treatment. Meanwhile, CeO_2_ is not found on the coating surface. This is probably because the content of CeO_2_ powder was very small and distributed on coating surface inhomogeneously during the cladding process. The corresponding atomic fractions (at. %) are: Al 50.35, C 10.47, Ti 9.14, O 21.04, and Fe 7.94. The atomic ratio of C to Ti is about 1:1 and constitutes the TiC phase. The atomic ratios of Al to O and Al to Fe are 3:2 and 3:1, respectively, due to the formation of the Al_2_O_3_ and AlFe_3_ phases. This is consistent with the XRD results shown on Figure 2. The main elements present in the coating are Al, C, Ti, and Fe. However, O impurities are present, the distribution of Al is uneven, and the three-layered shape and enrichment area can be seen on Figure 6c. This is mainly because at lower power, the diffusivity of Al is decreased, and as the molten pool temperature decreases, Al easily accumulates locally in the cladding layer, causing increased brittleness in the cladding layer. This can lead to cracks and pores. The elements C, Ti, and Fe are more uniformly distributed on the coating surface, as shown on Figure 6d–f. The Fe comes mainly from the substrate and coating diffusion layer.

**Figure 7:**
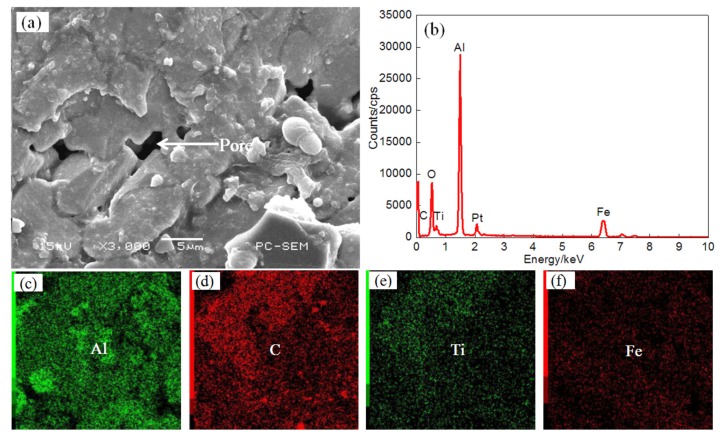
The surface SEM images (**a**) and EDS analysis result (**b**) and element content of Plane scan ((**c**) Al content (**d**) C content (**e**) Ti content (**f**) Fe content ) of Coating II.

Figure 7 shows the surface morphologies of coating II and the EDS plane scan analysis results when the laser power was increased to 1.2 kW. Short, rod-shaped structures can be seen on the surface of the coating, while the diameter of the pores is larger. The TiC reinforcing phase is less well distributed on the surface. The results of the EDS plane scan are shown on Figure 7b, and the corresponding atomic fractions (at. %) are: Al 47.96, C 10.88, Ti 9.96, O 21.54, and Fe 8.86. The atomic ratio of C to Ti is about 1:1 and constitutes the TiC phase. The main elements in the coating are Al, C, Ti, and Fe. In the coating, O is found as an impurity. Figure 7c shows that there is localized enrichment of Al on the surface of the coating, while the elements C and Ti are unevenly distributed on the surface, as shown on Figure 7d,e. When the power is increased, a part of the substrate enters the molten pool, so that the dilution rate of the coating is increased, and thus the reinforcing phase distribution is smaller per unit area. The Fe is uniformly distributed on the coating surface, as shown on Figure 7f.

**Figure 8:**
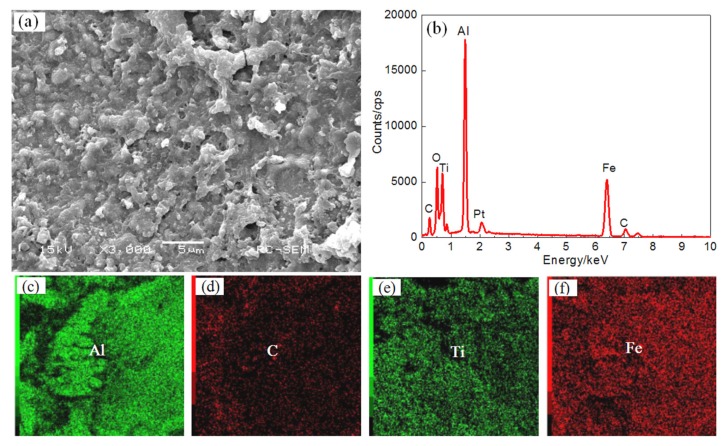
The surface SEM images (**a**) and EDS analysis result (**b**) and element content of Plane scan ((**c**) Al content (**d**) C content (**e**) Ti content (**f**) Fe content) of Coating III.

Figure 8 shows the surface morphology of coating III prepared with a laser power of 1.4 kW and the EDS plane scan analysis results. As shown on Figure 8a, the surface of the coating is flat and the pores have become smaller and smaller. The main structure of the coating is flaky or massive. The results of the coating EDS plane scan are shown on Figure 8b, and the corresponding atomic fractions (at. %) are: Al 34.21, C 11.30, Ti 11.25, O 29.57, and Fe 12.57. The main elements in the coating are Al, C, Ti, and Fe, while O is present as an impurity. The distribution of Al is uneven, as shown on Figure 8c. The elements C, Ti, and Fe are uniformly distributed on the surface, as shown on Figure 8d–f. The Fe content increased significantly. At greater laser power, melting the powder alloy takes less time, thus more substrate is involved in the reaction of the molten pool, increasing the interaction time with the substrate, making the melting between the components more complete. At the same time, the elements are more evenly distributed on the coating surface. This is also consistent with the results observed from the surface morphology of coating III.

**Figure 9:**
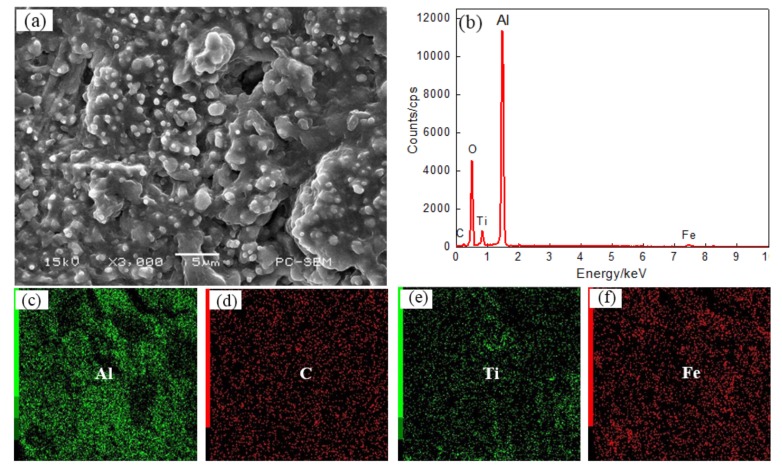
The surface SEM images (**a**) and EDS analysis result (**b**) and element content of Plane scan ((**c**) Al content (**d**) C content (**e**) Ti content (**f**) Fe content) of Coating IV.

The laser power was increased to 1.6 kW, and the resulting surface morphology of coating IV and the EDS plane scan analysis results are shown on Figure 9. The microstructure of the coating is small, granular, and massive and the structure has a tendency to connect and grow. The TiC reinforcing phase is evenly dispersed in the coating surface, as shown on Figure 9a. The results of the coating EDS plane scan are shown on Figure 9b, the corresponding atomic fractions (at. %) are: Al 32.71, C 12.08, Ti 11.94, O 29.50, and Fe 13.04. The main elements in the coating are Al, C, Ti and Fe, while O is present as an impurity. The distribution of the elements within the coating is more uniform, as shown on Figure 9c–f. As the laser power is increased, the components of the coating melt more fully, and the increased convection in the molten pool is more conducive to a uniform composition. On the other hand, an AlCe_3_ phase appears at high power, and AlCe_3_ is hexagonal system, with good fine grain strengthening, so that the structure is finer and more uniform.

In summary, with increasing laser power, the microstructure of the cladding layer changed from a lamellar and short, rod-like structure to massive and fine granular structure. The surface porosity of the cladding layer gradually decreased, while the surface flatness gradually increased. Because of the increased power, the energy absorbed per unit area of the cladding increases, thus the surface temperature of the cladding layer increases, the penetration depth increases, the surface temperature gradient changes, the cooling driving force decreases, and the cooling rate decreases. By reducing the temperature gradient in the cladding layer during crystallization, a more uniformly refined surface is obtained, improving cladding quality.

### 3.3. Microhardness Analysis 

**Figure 10:**
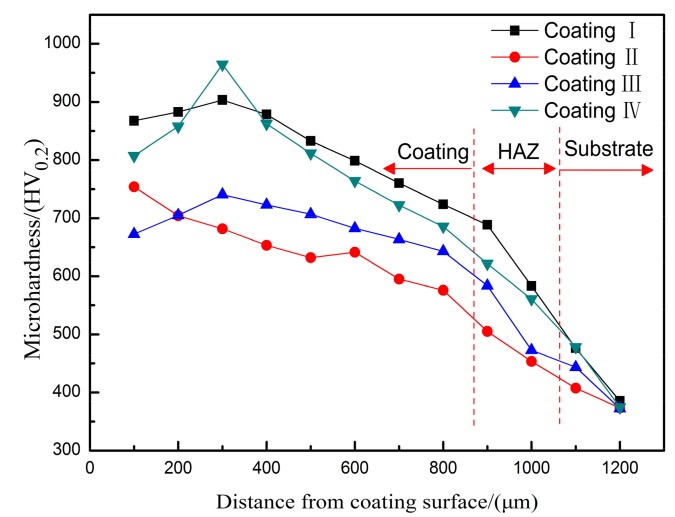
Microhardness profiles of the laser cladding intermetallic composite coatings under different laser power.

The microhardness distribution from the cladding surface to the substrate at different laser powers is shown on Figure 10. Compared with the substrate, the coating has a significantly increased microhardness; the maximum can be increased by about 3.5 times. At laser powers of 1 kW and 1.6 kW, the coating hardness is relatively high. At a laser power of 1.6 kW, the micro-hardness curve is relatively flat, and the microhardness reaches a maximum of 964.3 HV_0.2_ at a distance of about 300 μm from the surface because at higher powers, the depth and breadth of the melt pool become larger, the spray powder melts more fully, and the convective strength in the melt pool increases, resulting in a uniform distribution of TiC in the reinforcement phase with a morphology consisting of fine particles. At the same time, it can be seen from comparative XRD patterns that α-Al_2_O_3_ is contained in the cladding layer, which further enhances the hardness. At a power of 1 kW, the morphology of the cladding layer is uniform, but the distribution of the reinforcing phase TiC is more intensive. At 1.2 kW, the morphology of the reinforcing phase consists of short rods sparsely distributed in the cladding layer with a lower hardness. However, when the power is increased to 1.4 kW, the effect of fine grain strengthening is not obvious; the particles of reinforcing phase are larger and the surface of the cladding layer has more pores, which also weakens the microhardness to a certain extent. Therefore, with increasing laser power, the hardness of the cladding layer first decreases and then increases.

### 3.4. Friction and Wear Testing

**Figure 11:**
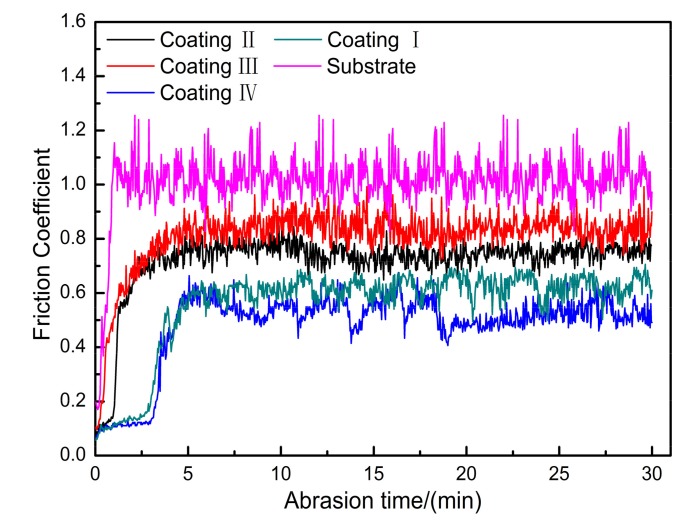
Friction coefficient of cladding layer with time.

**Figure 12:**
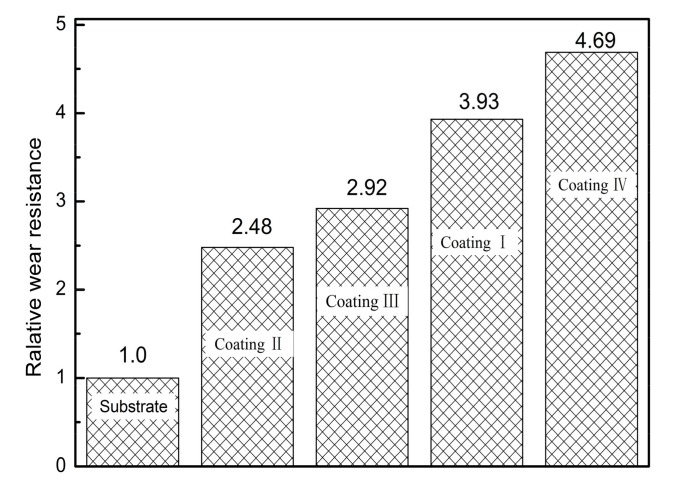
The relative wear resistance of cladding layer.

Figure 11 shows the air friction coefficient as a function of time for coatings prepared at different laser powers. It can be seen from Figure 10 that the friction coefficient increases rapidly to about 1.1 in ~0–3 min, then increases further before decreasing, and finally the friction coefficient stabilizes at about 1.2. This is because the substrate surface may form an oxide film. At first, the oxide film is not scratched and the friction increases gradually, so the friction coefficient also increases rapidly until the wear material, 45# steel scratched oxide film, and S355 steel come into direct contact, as the 45# steel hardness is HRC55, which is greater than that of the S355 steel substrate. Abrasive material, consisting of hard, abrasive particles relative to the substrate, can easily be drawn into the substrate by micro-cutting, so the friction coefficient will gradually decline and finally stabilize in a certain range. For the coatings formed at 1.2 and 1.4 kW laser power, it can be seen that the coefficient of friction first slowly increases to about 0.8 in the first few minutes and then fluctuates around 0.8 until the coefficient of friction stabilizes at around 0.7. Because the two coating surfaces contain unevenly distributed reinforcing phase TiC, the average hardness of the surface is better than that of the wear material, so during the wear process, the abrasive material may slightly cut into the cladding, and it is possible to produce scratches. With this wear and tear, the contact surface hardness is gradually reduced, the wear mode reverts to micro-cutting, and finally the coefficient of friction is gradually stabilized. For the coatings with laser powers of 1 kW and 1.6 kW, the surface of the cladding is smoother and its hardness is obviously higher than that of the wear-resistant material. Therefore, the wear mode of the cladding is mainly a small amount of scratching. Therefore, the friction coefficient curve fluctuates little, and finally stabilizes at about 0.5. Figure 12 shows the relative wear resistance of the cladding layers formed at different laser powers. It can be seen that the wear resistance of the cladding layer is obviously improved compared with that of the original substrate. The relative wear resistance of the cladding layers formed at 1 kW and 1.6 kW exhibited wear properties of 3.93 and 4.69, respectively, which is also consistent with the conclusion drawn from Figure 9. The reason why this phenomenon occurs is mainly because of the higher hardness of the coating at both powers, which showed good wear resistance [27].

### 3.5. Residual Stress and Cracks Analysis

**Figure 13:**
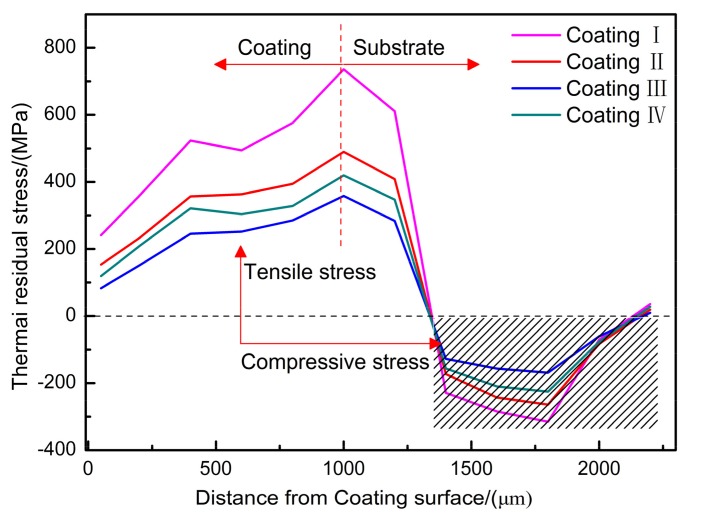
Residual stress distribution along the depth of coating: (**a**) Coating I; (**b**) Coating II; (**c**) Coating III; (**d**) Coating IV.

Figure 13 shows the longitudinal residual stress σ l along the depth of the claddings prepared at different powers. The longitudinal residual stress is parallel to the laser scanning direction. It can be seen from the results in the figure that the residual stress of each cladding layer is tensile stress. The surface residual stresses of coatings I, II, III and IV were 272.4 MPa, 153.3 MPa, 84.1 MPa, and 119.7 MPa. With increasing laser power, the residual stress on the coating surface first decreases and then increases. As the power increases, AlCe_3_ is precipitated into the cladding layer to further refine the crystal grains, and part of the substrate under the cladding layer undergoes annealing so that it absorbs a certain amount of residual stress during thermal cycling. Thus, the residual stress shows a downward trend, but with the further increase of power, the substrate melting and heating are increased, making the cladding layer retain a large residual stress when it is solidified. However, the changes in residual stress along the depth of the cladding are basically the same. When the distance from the cladding surface is about 1300 μm, the residual stress becomes compressive stress, because it is near the HAZ of the cladding layer and the substrate. The structure inevitably changes and the volume may change accordingly, causing the shrinkage of the cladding to be suppressed by the substrate and generating a tension on the cladding. Tensile stress corresponding to the edge of the substrate, by the force and reaction principle, forms a uniform compressive stress [28].

**Figure 14:**
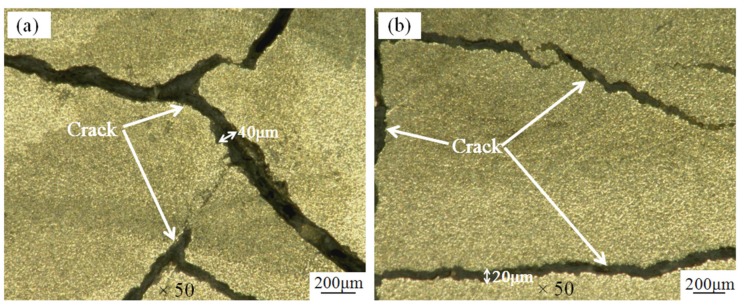

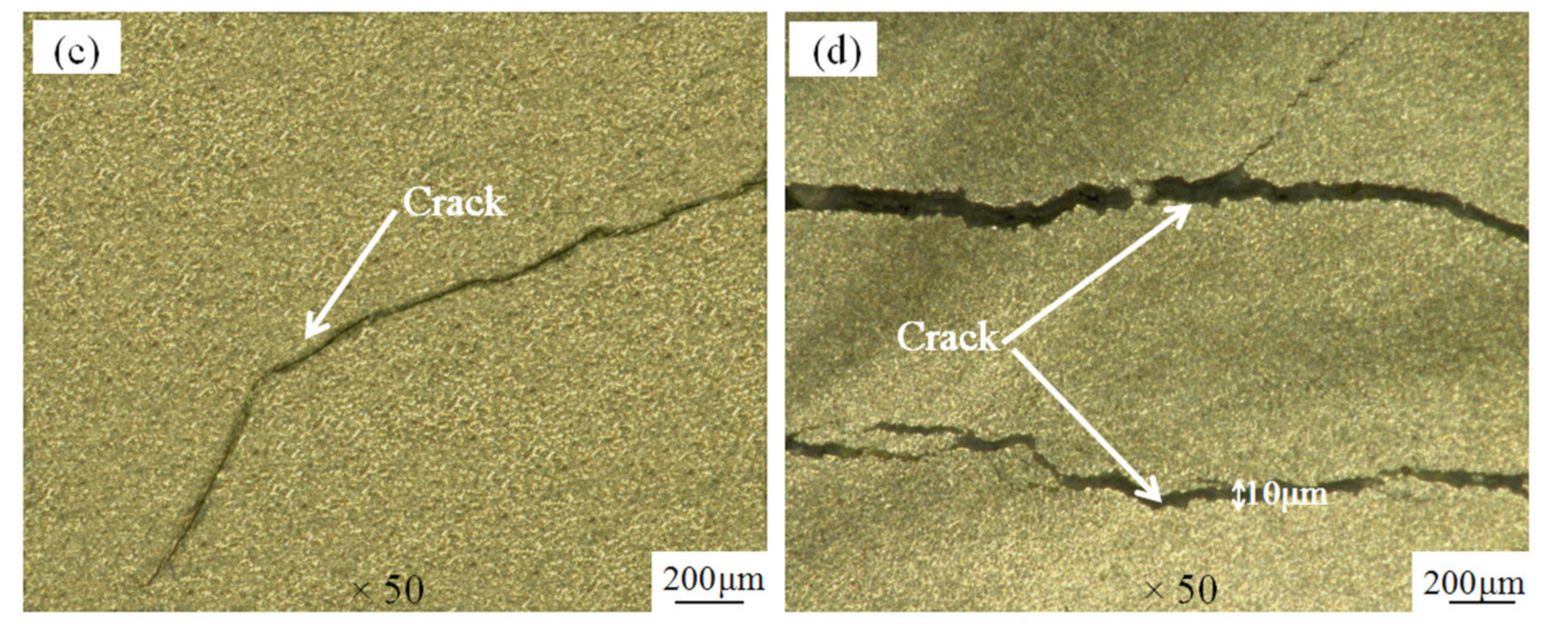
The surface low magnification morphology of the coating: (**a**) Coating I; (**b**) Coating II; (**c**) Coating III; (**d**) Coating IV.

Figure 14 shows a low magnification micrograph of a coating formed at low power. The surface cracks in coating I are wider and the number of cracks is greater, as shown on Figure 14a. When the power is increased to 1.2 kW, as shown on Figure 14b, the crack width of coating II is decreased but the number of cracks is still large. When the power reached 1.4 kW, the coating III surface cracks are relatively small and flat, as shown on Figure 14c; when the power continues to increase, the coating IV surface cracks gradually increased, but the crack width is small, as shown on Figure 14d. The crack rates of each coating were found to be 14 m^−1^, 10 m^−1^, 4 m^−1^, and 7 m^−1^. The dilution of the coating first decreases and then increases; different temperatures between the substrate and the cladding layer leads to the residual stress in the cladding layer first decreasing and then increasing. At the same time, while the coefficients of thermal expansion of the substrate and the cladding material are similar, the temperature of the cladding layer after solidification is higher than that of the substrate, so shrinkage of the cladding is larger than that of the substrate when cooled to normal temperature; the substrate is pressed and the cladding layer is pulled, resulting in the propagation of cracks [29].

**Figure 15:**
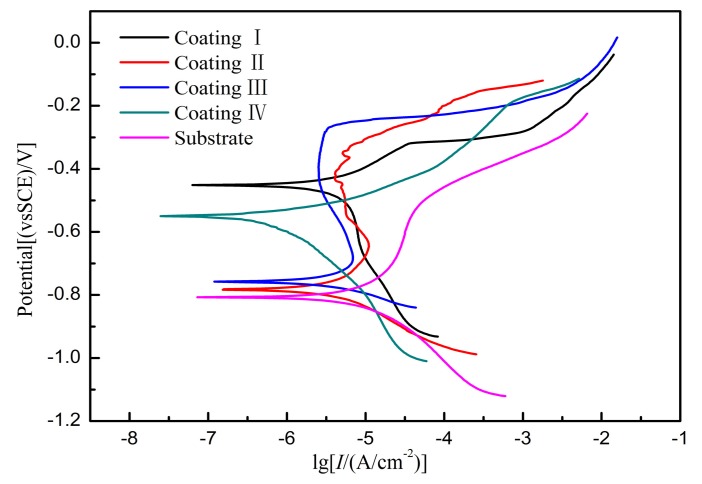
Potentiodynamic polarization of substrate and coatings with different powers.

Figure 15 shows the potentiodynamic polarization curves of the coatings and substrates prepared at different laser powers in a 5% NaCl solution. The corrosion potentials of coating II and coating III are both close to −0.8 V. When the corrosion potential ranges from −0.8 to −0.3 V, the curve clearly indicates a passivation phenomenon. Because of the deposition of a corrosive coating surface, the dissolution of coating corrosion is hindered. When the potential reaches −0.3 V, the passivation film on the coating surface is broken down, so that the corrosion current rapidly rises. When the laser power is 1.0 kW and 1.6 kW, the corrosion potentials of coating I and coating IV are close to −0.5 V. When the polarization potential exceeds −0.5 V, the current density increases, indicating that the oxide film on the surface of the coating is damaged due to anodic polarization, resulting in dissolution of the coating. The electrochemical parameters calculated from Tafel plots are listed in Table 3. The corrosion potential (*E_corr_*), corrosion current density (*I_corr_*), and the anodic/cathodic Tafel constants (*B_a_*, *B_c_*) were extracted directly from the potentiodynamic polarization curves by the Tafel fit method. The polarization resistance (*R_p_*) can be obtained from the Stern-Geary equation:

(2) $$R_{p}=\frac{B_{a}B_{c}}{\mathrm{2.303}I_{corr}(B_{a}+B_{c})}$$

and the corrosion speed (*V_corr_*) is defined as

(3) $$V_{corr}=\frac{MI_{corr}}{nF}$$

where *I_corr_* is the current density; *M* is the atomic weight of the metal; *n* is the valence of the metal; *F* is the Faraday constant.

As can be seen from the results of Table 3, from the corrosion kinetics point of view, *I_corr_*(IV) < *I_corr_*(I) < *I_corr_*(II) < *I_corr_*(III) < *I_corr_*(sub), and the corrosion speed was positively correlated with the current densities, but the polarization resistance was negatively correlated with the current densities. Generally, the values of *I_corr_*, *E_corr_*, and *R_p_* are important parameters for evaluating the corrosion properties of coatings. Lower *I_corr_*, positive *E_corr_*, and higher *R_p_* indicate that the coating provides better corrosion resistance [30]. Therefore, the corrosion resistances of the coatings were sorted as follows: coating IV > coating I > coating II > coating III. Compared with the bare substrate, the corrosion resistance has been significantly improved.

**Figure 16:**
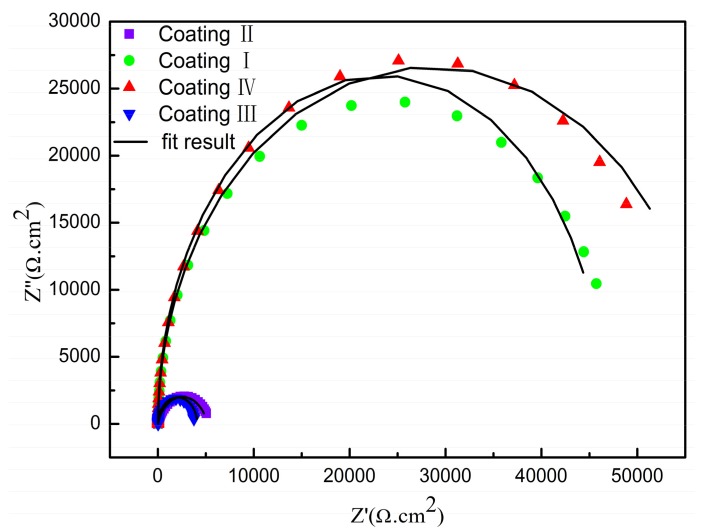
Nyquist of substrate and coatings with different powers.

**Figure 17:**
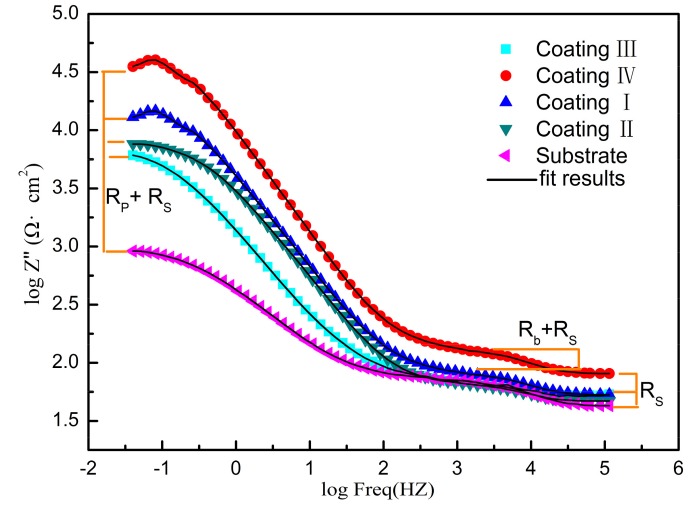
Bode plots of substrate and coatings with different powers.

**Figure 18:**
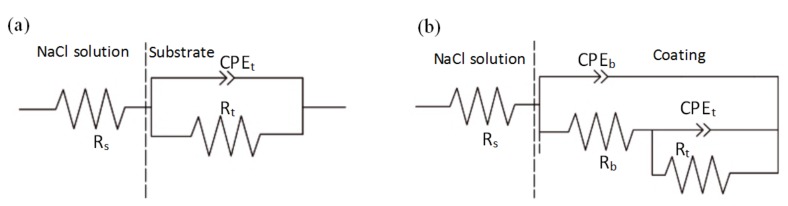
Equivalent circuits of the EIS plots for substrate (**a**) and coating (**b**) in the 5% NaCl solution.

Figure 16 is a Nyquist plot of the coating and substrate immersed for half an hour in a 5% NaCl solution, where Z’ is the real part of the impedance and Z” is the imaginary part. It can be seen that coating IV and coating I show a higher total impedance, and the maximum impedance of the coatings can reach about 25,000 Ω. In this case, a large capacitance arc occurs in the high frequency region, and the radius of the capacitor loop is large, so the corrosion resistance is better. Coating II and coating III exhibit capacitive arcs in the high-frequency region; the capacitive arc with a smaller radius corresponds to poor corrosion resistance. The substrate shows two capacitive arcs in the Nyquist plot, with a smaller radius at high frequencies and a larger radius at low frequencies, indicating that the substrate has stated to undergo pitting in solution [31]. Figure 17 shows a Bode plot of the substrate and coating. As can be seen, there are three platforms on the curve: the lowest frequency platform corresponds to the sum of the polarization resistance (*R_p_*) and the solution resistance (*R_s_*), the highest frequency platform corresponds to the solution resistance (*R_s_*), and the middle platform corresponds to the sum of the transfer resistance (*R_b_*) and the solution resistance (*R_s_*). It can be seen that the impedance of the substrate is 103 Ω·cm^2^ at low frequency. The impedance of the coating is one order of magnitude higher than that of the substrate, with the maximum impedance of coating IV being 104.5 Ω·cm^2^. According to the characteristics of electrochemical corrosion, the equivalent circuit shown on Figure 18 is used to model the results from impedance testing the substrate and coatings. Phase elements are characterized by Q and n, and the CPE is considered to be the ideal capacitance when the value of n is 1. In the equivalent circuit of Figure 18a, *R_s_* is the resistance of the NaCl solution and, *R_t_* is the resistance of the solution-substrate interface in parallel with CPEt. On Figure 18b, *R_s_* is the resistance of the NaCl solution, *R_b_* is the transfer resistance of the coating in parallel with the constant phase element CPEb, and *R_t_* is the barrier layer resistance of the coating in parallel with CPEt. ZSimpWin software was used to acquire EIS data for the substrate and coatings, and the corresponding equivalent circuit parameters are listed in Table 4. The values of *R_t_* for coatings I, II, III, and IV were 2.349 kΩ·cm^2^, 8.332 kΩ·cm^2^, 4.633 kΩ·cm^2^ and 10.348 kΩ·cm^2^, respectively, while the value of *R_t_* for the substrate was 0.685 kΩ·cm^2^. At the same time, the capacitance of the coating is obviously one order of magnitude lower than that of the substrate, which can improve the corrosion protection of the S355 Steel substrate. Compared with the other coatings, the corrosion resistance of coating IV is the best.

## 4. Conclusions

(1) A more compact Al-TiC-CeO_2_ composite coating was prepared by laser cladding technology, and the dilution rate of the coating was less than 10%, showing a good metallurgical bond with the substrate. With increasing laser power, the microstructure of the coating changes from massive and short rod-like to fine granular, and the fine grain strengthening effect is obvious. Elements are evenly distributed within the coating.
(2) When the laser power was 1 kW and 1.6 kW, the microhardness of the resulting coatings was relatively high, and the microhardness changes relatively smoothly. The microhardness can reach 964.3 HV_0.2_ at a depth of about 300 μm from the surface. The abrasion resistance of the coating first decreases and then increases with increasing laser power. At a power of 1.6 kW, the relative wear resistance of the cladding was 4.69 and the wear resistance was improved.
(3) The residual stress in the coating surface is tensile stress. With the increase in laser power, the residual stress of the coating surface first decreases and then increases due to the changing temperature difference between the substrate and the cladding layer, resulting in cracks.
(4) The corrosion resistances of the coatings were sorted as follows: coating IV > coating I > coating II > coating III. Compared with the bare substrate, the corrosion resistance has been significantly improved.

## Figures and Tables

**Table 1:** Chemical composition of S355 steel (wt. %).

C	Si	Mn	P	Cr	S	Ni	Mo	Zr	Fe
0.17	0.55	0.94	0.035	0.065	0.035	0.065	0.30	0.15	97.69

**Table 2:** Laser cladding process parameters.

Parameters	Values
laser power/[W]	1000, 1200, 1400, 1600
laser scanning rate/[mm·min^−1^]	420
Powder feeding rate/[g·min^−1^]	8
Argon gas velocity/[L·min^−1^]	15
Spot diameter/[mm]	3

**Table 3:** Electrochemical data of substrate and coatings with different powers.

Sample	*E*_corr_	*I*_corr_ (A/cm^2^)	*B*_*a*_ (mv)	*B*_*c*_ (mv)	*R*_*p*_ (Ω·cm^2^)	Corrosion Rate (mm·a^−1^)
Coating I	−0.45174	1.091 × 10^−6^	202.57	340	13,556.2	0.0084898
Coating II	−0.80756	5.5547 × 10^−6^	350.36	154.67	6458.6	0.043226
Coating III	−0.79293	7.6735 × 10^−6^	91.96	107.32	5193.5	0.059715
Coating IV	−0.55062	6.2217 × 10^−7^	68.5	182.83	30543	0.0048417
Substrate	−0.80172	2.7706 × 10^−5^	108.21	314.51	1294.7	0.2156

**Table 4:** EIS data of substrate and coating.

Sample	R_s_	Q_b_	N_b_	R_b_	Q_t_	N_t_	R_t_
	(Ω·cm^2^)	(Ω^−1^·s^−n^·cm^−2^)		(kΩ·cm^2^)	(Ω^−1^·s^−n^·cm^−2^)		(kΩ·cm^2^)
Substrate	16.26	−	−	−	1.069 × 10^−3^	0.8	0.685
Coating I	12.63	1.949 × 10^−4^	0.8912	5.83	5.325 × 10^−4^	0.8533	2.349
Coating II	12.91	2.417 × 10^−6^	1	10.84	4.433 × 10^−4^	0.8523	8.332
Coating III	11.76	4.175 × 10^−6^	0.9998	6.554	1.985 × 10^−4^	0.8369	4.633
Coating IV	11.11	5.355 × 10^−6^	1	4.817	2.083 × 10^−4^	0.8417	10.348
